# Exploring the Synergistic Potential of Radiomics and Laboratory Biomarkers for Enhanced Identification of Vulnerable COVID-19 Patients

**DOI:** 10.3390/microorganisms11071740

**Published:** 2023-07-03

**Authors:** Catharina Gerhards, Verena Haselmann, Samuel F. Schaible, Volker Ast, Maximilian Kittel, Manfred Thiel, Alexander Hertel, Stefan O. Schoenberg, Michael Neumaier, Matthias F. Froelich

**Affiliations:** 1 Institute for Clinical Chemistry, Medical Faculty Mannheim of the University of Heidelberg, Theodor Kutzer Ufer 1-3, 68167 Mannheim, Germany; 2 Department of Radiology and Nuclear Medicine, University Medical Center Mannheim, Medical Faculty Mannheim of the University of Heidelberg, Theodor-Kutzer-Ufer 1-3, 68167 Mannheim, Germany; 3 Department of Anaesthesiology and Surgical Intensive Care Medicine, Medical Faculty Mannheim of the University of Heidelberg, Theodor-Kutzer-Ufer 1-3, 68167 Mannheim, Germany

**Keywords:** COVID-19, SARS-CoV-2, coronavirus infection, integrative medicine, intensive care units, thoracic radiography, cell-free nucleic acid, algorithms

## Abstract

Background: Severe courses and high hospitalization rates were ubiquitous during the first pandemic SARS-CoV-2 waves. Thus, we aimed to examine whether integrative diagnostics may aid in identifying vulnerable patients using crucial data and materials obtained from COVID-19 patients hospitalized between 2020 and 2021 (*n* = 52). Accordingly, we investigated the potential of laboratory biomarkers, specifically the dynamic cell decay marker cell-free DNA and radiomics features extracted from chest CT. Methods: Separate forward and backward feature selection was conducted for linear regression with the Intensive-Care-Unit (ICU) period as the initial target. Three-fold cross-validation was performed, and collinear parameters were reduced. The model was adapted to a logistic regression approach and verified in a validation naïve subset to avoid overfitting. Results: The adapted integrated model classifying patients into “ICU/no ICU demand” comprises six radiomics and seven laboratory biomarkers. The models’ accuracy was 0.54 for radiomics, 0.47 for cfDNA, 0.74 for routine laboratory, and 0.87 for the combined model with an AUC of 0.91. Conclusion: The combined model performed superior to the individual models. Thus, integrating radiomics and laboratory data shows synergistic potential to aid clinic decision-making in COVID-19 patients. Under the need for evaluation in larger cohorts, including patients with other SARS-CoV-2 variants, the identified parameters might contribute to the triage of COVID-19 patients.

## 1. Introduction

The pandemic spread of severe acute respiratory syndrome coronavirus type 2 (SARS-CoV-2) and the emergence of coronavirus disease 2019 (COVID-19) has had enormous global health and socio-economic consequences and high infectivity as well as hospitalization rates have put hospital bed and Intensive-Care-Unit (ICU) capacities under enormous stress during the first pandemic waves [1]. Accordingly, the rapid identification of disease severity enabling triaging of patients is an essential clinical aspect and requires a multidisciplinary approach to optimize the diagnostic potential. Various routine laboratory parameters associated with disease severity have already been described, but an integrative approach including Radiomics and cfDNA is missing so far. Among those, C-reactive protein (CRP), activated partial thromboplastin time (PTT), D-dimer, and lactate dehydrogenase (LDH) have been reported [2,3,4]. Moreover, previous studies have shown an association between increased cell-free DNA (cfDNA) and a severe course of COVID-19 [5,6]. In this study, we intended to identify suitable markers associated with intensive care requirements. The routine laboratory parameters examined in other studies were further augmented by quantified cfDNA in our work as a dynamic marker of cell decay. Since we anticipated increased cell death of lung tissue, especially in the presence of lung consolidations, this study was called “Laboratory Assessment of Ground Glass Opacities” (LAGGO), emphasizing the interdisciplinary aspect of the work.

While blood-bourne laboratory parameters serve as surrogate markers for monitoring various organ functions, the chest’s computed tomography (CT) adds important diagnostic topological information on lung involvement in COVID-19 patients [7]. Tsang et al. have developed the SARS severity score to estimate the severity of lung involvement semi-quantitatively [8]. Additionally, the Radiological Society of North America has developed a structured reporting system that classifies findings related to COVID-19 [9]. Radomics analysis of COVID-19 CTs aims to quantify lung involvement in a fully automatic and reader-independent fashion. Thanks to recent advances in deep-learning-based machine vision, the software can aid the image segmentation necessary for radiomics analyses [10]. Radiomics is an innovative and rapidly evolving field, including the extraction and analysis of quantitative features from medical imaging. By converting an image into mineable data, radiomics complements the traditional visual interpretation and enables a quantitative evaluation of radiological images. In this manner, radiological data can be leveraged not only for qualitative evaluation but also in the form of diverse quantitative datasets to enable personalized patient predictions. This presents many opportunities for analysing radiological data, particularly in assessing tumor diseases, where it is commonly applied. However, ongoing research is necessary to prove the promising potential of radiomics with regard to acquisition protocols, segmentations, and feature extractions [11].

Therefore, so far, the use of radiomics is not widely adopted in the clinical setting, yet [12]. Both laboratory medicine and radiology provide complementary diagnostic value in various stages of COVID-19. Thus, we investigated the potential value of integrated diagnostics in estimating the likelihood of ICU admission to aid in planning ICU capacities in managing Corona cases.

For this purpose, we utilized conserved residual specimens obtained during the initial SARS-CoV-2 pandemic outbreaks to quantify cfDNA and reanalyzed previously acquired data to retrospectively evaluate the significance of specific biomarkers in predicting a severe hospitalized COVID-19 course. Thus, in this study, we present biomarkers that potentially allow discrimination between ICU requirements and normal inpatient treatment in cases of infection with the first SARS-CoV-2 variants in Germany.

The primary objective of this investigation is to establish a suitable algorithm for identifying distinct laboratory and radiology parameters correlated with the need for intensive care unit (ICU) admission (aim I). Subsequently, a verification of the selected parameters via an alternative method is required (aim II). Furthermore, in case of a substantial number of parameters, selecting the most significant ones has to be performed via an algorithm (aim III). Finally, the individual radiomics, RSNA Score, routine laboratory, cfDNA and combined variables have to be compared in their predictive power (aim IV).

## 2. Materials and Methods

### 2.1. Participant Recruitment

**Figure 1:**
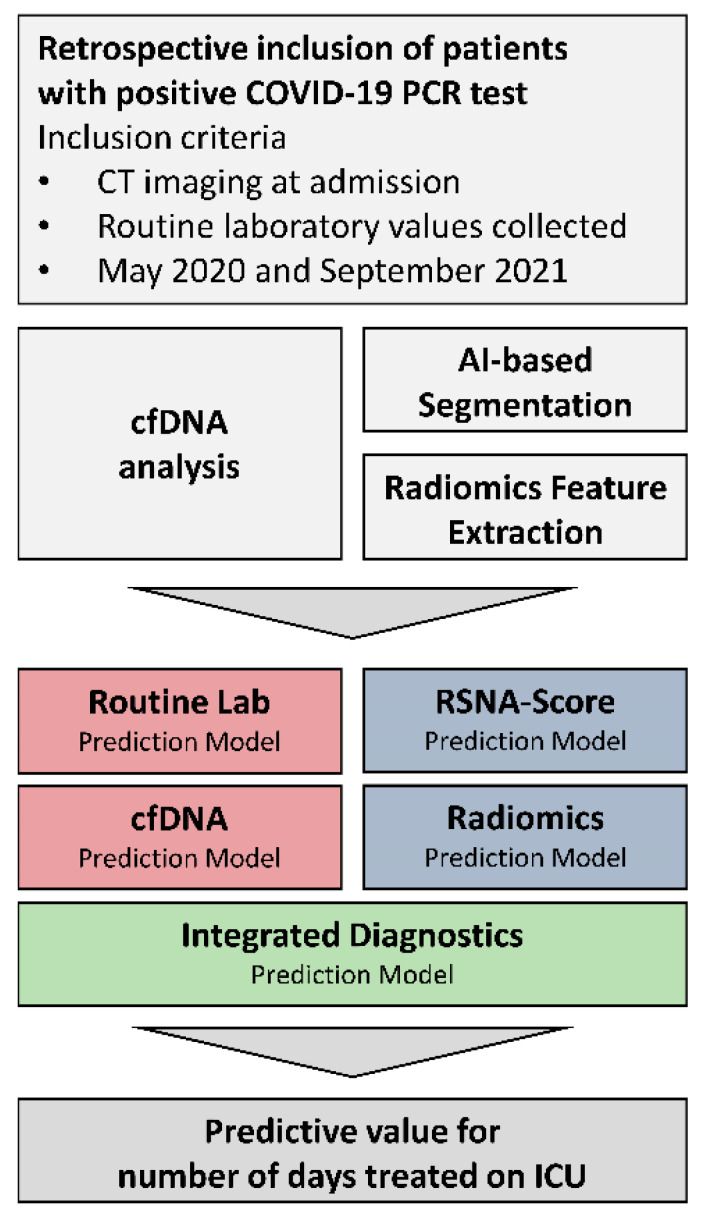
Study concept.

From May 2020 to September 2021, SARS-CoV-2 patients aged 18 or older previously confirmed by qPCR were enrolled in the LAGGO (Laboratory Assessment of Ground Glass Opacities) study at the University Medical Center Mannheim, Germany (see Figure 1). Informed written consent was obtained from each subject (*n* = 52). The Institutional Review Board (2020-541N) approved the study protocol, and the study was conducted in accordance with the Declaration of Helsinki. During the initial wave of the SARS-CoV-2 pandemic, we deemed it inappropriate to obtain informed consent when requiring intensive care treatment based on ethical considerations. Therefore, the study inclusion was conducted retrospectively after the completion of treatment. Considering this aspect and the high mortality rate, this accounts for the limited number of participants. We have to address this point in the study’s limitations. 

Figure 1: Presentation of the study concept and the research objectives. The inclusion criterion in the study was the diagnosis of COVID-19 based on a positive qRT-PCR result of a nasopharyngeal swab. Radiological chest CT data were segmented and radiomically analyzed. In addition, the patient’s routine laboratory was evaluated, and cfDNA was prospectively isolated and quantified. Radiological and laboratory features were selected separately for predicting the duration of intensive care. Before inclusion in an integrated prediction model, the existence of collinearities was reduced using a minimal redundancy algorithm. The final model intends to indicate the patient outcome by predicting an intensive care requirement and facilitating clinical decisions.

### 2.2. Routine Laboratory Analysis

Blood count was measured on Sysmex XN-9000 (Sysmex, Hamburg, Germany) platform. Hemostaseological parameters were determined on the CS-5100 analyzer (Sysmex, Hamburg, Germany). Clinical chemistry biomarkers were measured on an Atellica-CH Analyzer (Siemens Healthcare GmbH, Eschborn, Germany). For all measurements, the dedicated reagent systems were used according to the manufacturers’ recommendations and after internal verification in compliance with DIN EN ISO 15189 in an accredited laboratory. Pre-analytical quality was subsequently judged by centrifugation using the hemolysis assessment system of the analyzer platform on an ordinal scale ranging from no (0) to significant hemolysis (5)). For samples exceeding the value “1”, the results for LDH and ASAT were not used in the respective samples since an influence with regard to increased values is described [13,14]. Although the manufacturer does not specify any restrictions in the corresponding instructions, we decided to enhance the quality of the preanalytic by the mentioned procedure. Blood gas analyses (BGA) from arterial and venous blood were conducted under point-of-care-testing conditions.

### 2.3. Sample Collection and cfDNA Analysis

For the isolation of cfDNA, ethylene diamine tetraacetic acid (EDTA) plasma obtained when clinically indicated was processed within 4 h of blood collection. Specimens were centrifuged at 1600× *g* for 10 min at 20 °C. The supernatant was transferred to a new 15 mL tube and centrifuged at 3000× *g* for 10 min. Optical control for hemolysis was performed, and insofar as it was visually detectable, the sample was excluded. The final supernatant was stored at −80 °C until the isolation of the cfDNA. CfDNA was isolated using the Qiagen QIAmp Circulating Nucleic Acid Kit (Qiagen, Hilden, Germany) according to the manufacturer’s instructions without modifications. For cfDNA isolation, the maximal plasma volume processed from the subject’s specimen was utilized (range 0.4 and 1.5 mL). The quantification of the cfDNA was performed by means of a Qubit Fluorometer and Qubit cfDNA HS Assay Kit (Invitrogen, Los Angeles, CA, USA) and the results was normalized via a control with known concentration included in each measurement. In addition, the determined concentration was recalculated in relation to the input volume and reported as ng per mL plasma.

### 2.4. Chest CT Imaging

All patients in this study underwent native or contrast-enhanced CT imaging of the chest. The scans were performed on either a SOMATOM Definition AS, SOMATOM Definition Flash or a SOMATOM Definition 64 (Siemens Healthcare GmbH, Erlangen, Germany). Depending on the history, clinical presentation and possible comorbidities, patients were scanned using one of the following protocols: Low-dose CT, routine non-contrast-enhanced CT, contrast-enhanced CT or CT pulmonary angiography. In total, 74.54% of scans were performed with contrast agents, of which 58.54% were performed as arterial phase CT. Imeron 300 (Bracco Imaging S.p.A., Milan, Italy) was used as a contrast agent in a dose adjusted for CT protocol and weight. 

### 2.5. Chest CT Imaging Analysis

CTs were analyzed by a resident radiologist, using a semi-quantitative score to quantify pathological changes in the lung parenchyma. To calculate the score, each lung is divided into three sections and scored from 0–4 with regard to severity. For 25% involvement, one point is given per section. Then the sum of all six sections is added, resulting in a score from 0 to 24 [9]. Furthermore, CTs were analyzed quantitatively with radiomics methods using the research application MM Radiomics Frontier Prototype 1.2.6. (August 2016, Siemens Healthcare GmbH, Erlangen, Germany) within syngo.via VB60A (May 2021, Siemens Healthcare GmbH, Erlangen, Germany). To extract radiomics features, segmentation of CT scans is necessary [13]. Segmentation was collected in an automated fashion using the deep-learning-based research segmentation application CT Pneumonia Analysis prototype 2.5.2 (April 2021, Siemens Healthcare GmbH, Erlangen, Germany). This software is currently classified as “for research use only”. A binWidth of 25, a 512 × 512 matrix, voxelArrayShift of 0 was applied. For the analysis, pyradiomics version 2.1.0 was applied. Only original radiomics features were included in the analysis.

### 2.6. Performance of Feature Selection and Statistical Analysis

For identifying adequate parameters associated with a severe course, we opted for an algorithm-based training of a model. A multivariate linear regression with internal 3-fold cross-validation was performed to construct a linear model initially predicting the duration of intensive care in days. It was adapted into a categorizing model dividing subjects into “ICU” versus “no-ICU-demand”. This was realized separately for laboratory and radiological data based on a stepwise forward and backward feature selection to create a linear regression model with “ICU period in days” as the initial training target. With regard to the routine laboratory, all mentioned parameters exclusive to cfDNA were used for internal cross-validation comprising three sub-datasets randomly split (each consisting of *n* = 22 for the training and *n* = 10 for the validation). The training was always performed on 22 subjects and validated in the unaffected cohort. This was repeated successively with different cohort formations to obtain a more representative selection despite the limited number of participants. In addition, we used two selection methods-forward and backward selection. The forward selection is a method using subsets of features to train the model, starting with one variable, and adding further variables in each iteration until no model improvement can be achieved. Regarding backward selection all parameters are used initially and then reduced until the model deteriorates due to the omission of variables. 

Due to the high number of variables identified, especially for Radiomics, a further reduction before integration into a model was essential. Therefore, a ranking was implemented via the frequencies of feature selection in the sub-datasets resulting in values between 0–3 (0: not selected in a sub-dataset; 3: selected in all three datasets). We excluded parameters selected only once or less.

Moreover, we used a random forest algorithm to verify the selected laboratory parameters and to examine the relevance of cfDNA predicting the regression target “ICU period”. The algorithm creates shadow variables for each real variable by permutation and compares the importance of the real variable with the maximum importance of all shadow variables. If the real variable shows higher importance than the corresponding shadow variable, the algorithm assigns high importance to the feature [14,15]. The feature selection was performed identically for the radiological parameters. 

Furthermore, we first created separate correlation plots for radiomics and laboratory data in R Studio to identify collinearities using the “library(corrplot)”. Subsequently, we applied a minimal redundancy algorithm utilizing the following commands, among others “findCorrelation”, “library(heatmap)”) to reduce redundant parameters as a combination of collinear variables would not enhance the predictive potential. The selection of initial parameters, including clustering of strongly correlated variables (shown in dark brown), is presented as the first correlation plot in the results. Following the reduction of parameters using the algorithm, a second visualization in the form of a correlation plot is provided. These parameters were then used for singular radiomics or laboratory models and the integrative model.

After the variable reduction, the maintaining potential to classify subjects was illustrated by a heatmap performing unsupervised clustering based on the final parameters (R package “pheatmap”). The application of the validation dataset served to prove the maintenance of classification potential and not to determine the model’s power, as this would lead to overfitting (Supplemental Material). Due to the Root Mean Square Error (RMSE) of predicted and actual days in ICU, even in our validation cohort, the model was adapted to a logistic regression approach with the clinical decision endpoint “ICU stay yes/no” and a cut-off for this categorization has been selected based on this RMSE. 

The final verification and the determination of the accuracies of the integrative model were realized with a training and validation independent test cohort. In addition to establishing an integrative model, we compared individual cfDNA, RSNA score, radiomics or routine laboratory models with the combined model. The prediction of ICU needs was performed using the test cohort in R-Studio. To accomplish this, we applied the previously trained and validated models on the test cohort as a logistic model. The algorithm employed classified values above six as indicating “ICU need” and values below six as indicating “regular inpatient treatment”.

Additionally, we conducted a ROC analysis to compare true positives with false positives based on the test cohort (“library(ROCR)”). This analysis was performed for different models, and the Area under the Curve (AUC) was calculated. Patient’s symptoms were not included in the model but compared between ICU and non-ICU cohorts. All statistical analyses, including comparing demographics, COVID-19 symptoms, treatment and laboratory parameters of ICU and non-ICU cohorts, were performed using R statistics software (Version 4.1.2) [16]. Cohort comparisons of non-normally distributed continuous variables were performed by the Kruskal-Wallis rank sum test, and normally distributed continuous variables were compared via regular ANOVA test. Categorical variables are presented as frequency and percentage. For the comparison of categorical variables, a Fisher exact test was performed. *p*-values <0.05 were considered significant.

## 3. Results

### 3.1. Demographics and Clinical Aspects

**Figure 2:**
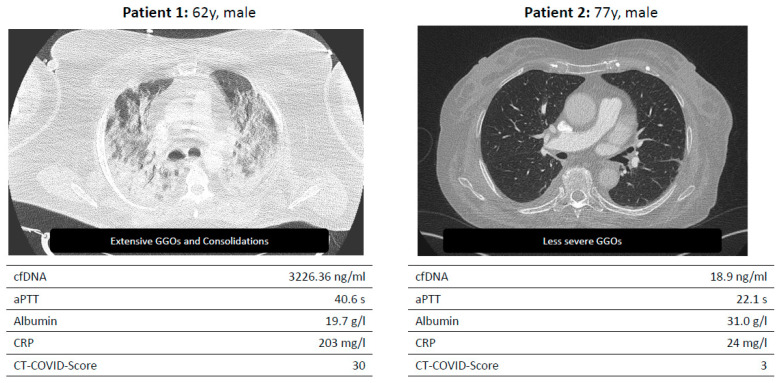
Two example patients enrolled in the LAGGO study.

For the assessment of the diversity of the disease in COVID-19 severity and treatment, a comparison between the ICU- and non-ICU cohorts was performed. Moreover, this comparison revealed significantly elevated laboratory parameters in cases requiring ICU admission (Table 1/Figure 2). Participants in whom CT could be assessed for pulmonary embolism were not observed to have a central or distal embolism.

Exemplary presentation of two test persons with severe and mild progression.

### 3.2. Prognostic Value of Laboratory Parameters

#### Creation of the Laboratory Prediction Model

Differences in laboratory parameters between the ICU and non-ICU cohorts are summarised in Table 1. Moreover, the training and cross-validation described in more detail in the methods were performed with a dataset comprising three sub-datasets (Table 2). The most frequent parameters, PTT, albumin, GGT and CRP, and ALT, platelets, and WBC, were selected in two training sets and were included in further analysis. 

**Figure 3:**
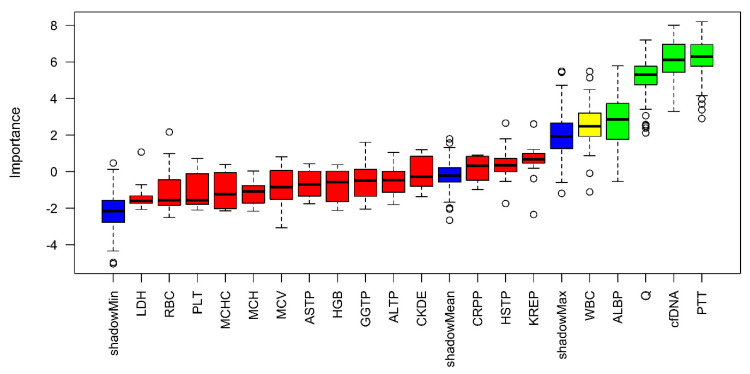
Random–Forest Approach estimating the variable importance for predicting ICU days.

In addition, the feature selection was methodically verified using a random forest analysis with the same target as our regression model (ICU stay). This was done to verify the importance of the variables selected by forward and backward feature selection. In the following, the previously selected parameters were used, but due to the high importance of cfDNA, cfDNA was included in the further establishment of the prediction model (Figure 3).

High importance is illustrated by green, medium by yellow and low by red. In addition, the minimum, mean, and maximum importance of the shadow variables are shown in blue. Parameters with a lower relevance for predicting the duration of intensive care requirements than the maximum shadow variable have been assigned low importance.

Furthermore, we considered the first BGA results of the subjects and examined the results. The parameters were investigated for their suitability as predictors of ICU admission via unsupervised clustering in Supplemental S1. Here, no clear differentiation between normal inpatients to long–term intensive care patients could be observed as the values were either similar among the groups (see pH variation) or showed heterogeneities within all subcohorts.

### 3.3. Prognostic Value of Radiological Parameters

#### Creation of the Radiological Prediction Model

The radiomics data were equally cross − validated (*n* = 30), and the ranking was performed equivalently as previously described. Due to the high diversity of radiomics, the algorithm selected more parameters per dataset than for the laboratory data. Details of all identified parameters of each sub-dataset are presented in Table 2. 16 parameters were selected for establishing a model predicting ICU stay (original_firstorder_10Percentile, original_gldm_LargeDependenceLowGrayLevelEmphasis, original_shape_Maximum2DDiameterSlice, original_firstorder_Energy, original_firstorder_TotalEnergy, original_glcm_ClusterShade, original_glcm_DifferenceVariance, original_glrlm_RunEntropy, original_glrlm_RunLengthNonUniformity, original_ngtdm_Busyness, original_ngtdm_Contrast, original_shape_Elongation, original_shape_Flatness, original_shape_LeastAxisLength, original_shape_MajorAxisLength, original_shape_Maximum3DDiameter). Therefore, a reduction of the selected parameters was essential, as described in the following.

In addition, the CT COVID Severity (RSNA) score was used as a variable to predict ICU stay.

### 3.4. Prognostic Value of Integrated Diagnostics

**Figure 4:**
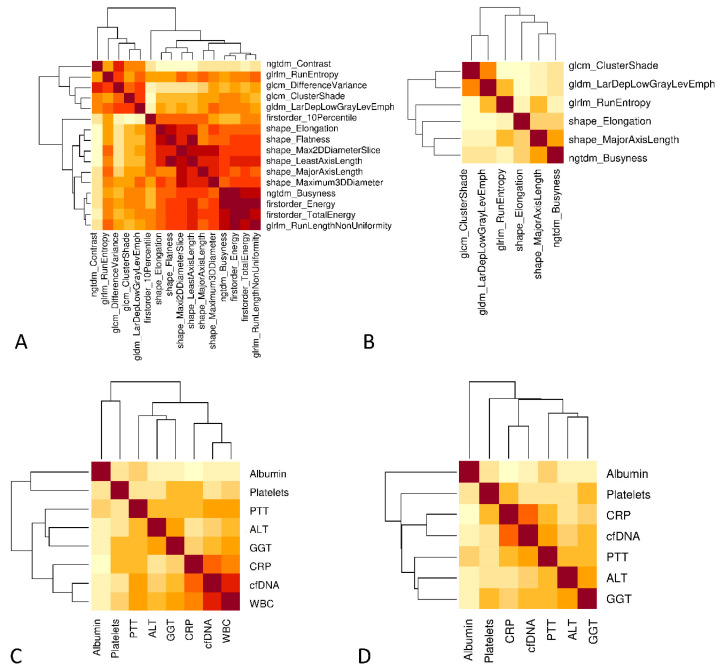
Reduction of collinearity. (**A**) Correlation plot to identify potential clusters of radiomics. The degree of correlation is classified by the brightness of the colors (dark brown corresponds to a high correlation). (**B**) Clustering was reduced by the use of a minimal redundancy algorithm. (**C**) Correlation plot of laboratory parameters. (**D**) Exclusion of “white blood cells” because of the highest correlation with CRP. (**B**,**D**) Variables were included in the final integrative model.

The radiological and laboratory parameters were examined for collinearities before integration into the final models, as a reduction of features was essential. Since various correlations were identified, we applied a redundancy reduction algorithm (Figure 4). For this purpose, separate correlation matrices were initially created for radiomic parameters (Figure 4A) and laboratory parameters (Figure 4C). A high correlation between parameters is illustrated by a dark color. After applying the “findCorrelation” command, which identifies collinear parameters and removes one of the two variables, updated correlation plots were generated for the remaining radiomic parameters (Figure 4B) and laboratory parameters (Figure 4D). Finally, seven laboratory (albumin, ALT, GGT, platelets, PTT, CRP and cfDNA) and six radiomics parameters (original_glcm_CLusterShade, original_gldm_LargeDependenceLowGrayLevelEmphasis, original_glrlm_RunEntropy, original_shape_Elongation, original_shape_MajorAxisLength and original_ngtdm_Busyness) were integrated in the combined model. 

After successfully identifying suitable parameters, we created a heatmap illustrating the unsupervised clustering of the combined dataset. We applied the model to our cross-validation dataset (Supplemental S1) to verify the selected variables even after the previously described variable reduction. 

**Figure 5:**
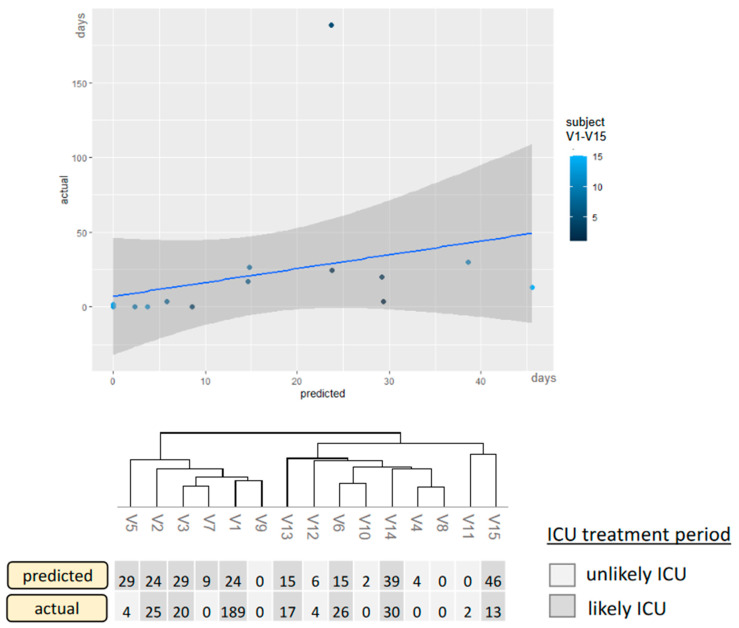
The integrative model applied to the validation-independent test set.

Moreover, the period in ICU predicted by the integrative model was compared to the actual days in an independent test cohort (*n* = 15, Figure 5). The Root Mean Square (RMSE) of the deviations between the actual and predicted days was 5.3 days in the cross-validation set and 12.3 days in the test-cohort set (outliner V5 is excluded as the training set is not representative of values above 40 days). The application on the validation cohort only served to verify the variable selection even after reducing the initial parameters and not to assess the model’s power, as this would cause overfitting. Based on these results, revealing limitations in the linear prediction of shorter ICU stay even in the validation cohort, the linear approach had to be adapted via a categorization into likely ICU and unlikely ICU with six days as a decision cut-off between intensive care and normal care treatment. 

Correlation between actual and predicted ICU treatment applied to a second dataset not affected by cross-validation. The predicted days are compared to the actual days, and the model is categorized as described previously. X-axis: predicted ICU days, y-axis: actual ICU days for the validation-naïve patients V1–V15. Only the categorizing version represents the final model. Thus, the light-colored patients would have a recommendation for normal inpatient treatment, and the dark-colored patients would have a referral for ICU treatment.

**Figure 6:**
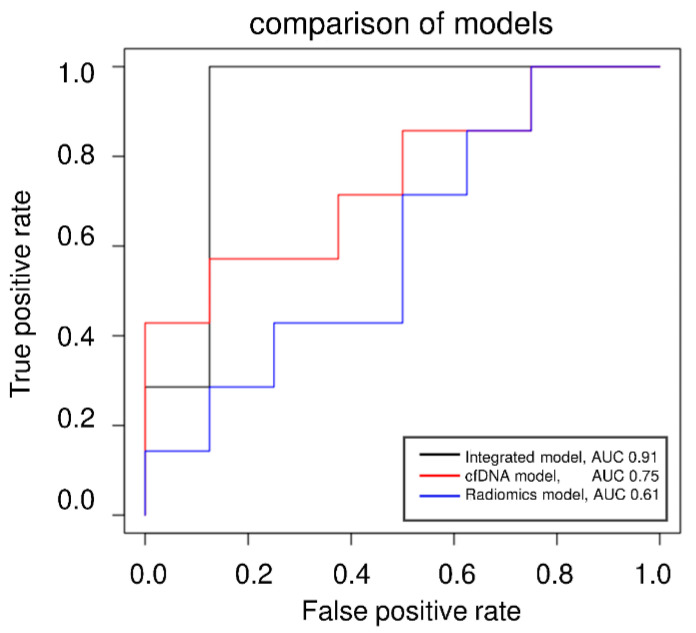
ROC analysis of the categorized approach.

The cut-off was based on the RMSE in the validation set and was finally tested in a validation-independent cohort. Two false positives were identified in the test cohort resulting in an AUC of 0.91 in ROC analysis (Figure 6). Compared to singular models (RSNA, cfDNA, Radiomics, Routine lab), the integrated model demonstrates the highest predictive potency for intensive care requirements (accuracy = 0.87, Table 3). The accuracies were determined using the independent test set not used for prior cross-validation. 

ROC analysis of the cfDNA model, the Radiomics model, and the integrated model applying the categorized approach predicting ICU demand (yes/no).

## 4. Discussion

This study assessed several diagnostic models for predicting the requirement of intensive care treatment in COVID-19 patients. The special aspect of our model is the integration of routine laboratory, cfDNA, and radiomics, which was intended to increase the diagnostic potential and has thus been trained, validated, and verified in independent datasets. Our results show a synergistic potential of laboratory and radiological parameters to support clinical decision-making in COVID-19 patients.

These results align with published literature but gain additional insights using a truly interdisciplinary diagnostic assessment. Some studies have focused on differentiating COVID-19 pneumonia from other lung conditions [17]. Subsequently, the prognostic value of radiomics based on initial CT scans was investigated by Zhang et al., who proposed an AI-based radiomics nomogram to predict disease progression in COVID-19 patients [18]. Similarly, Lassau et al. have shown via AI-deep-learning mechanisms that the severity of COVID-19 can be predicted by integrating CT scan data and biological and clinical parameters [19]. Some other studies have also dealt with outcome parameters such as hospitalization, patient management, or organ involvement, such as acute renal failure in COVID-19.

In some cases, as in our work, cell decay markers (LDH instead of cfDNA), acute phase parameters (CRP, WBC), and quantitative lung parenchyma data were identified as possible predictors. Thus, the selection of our potential predictors is partially supported by results published in other studies [20,21,22]. Nevertheless, combining radiomics, routine laboratory, and cfDNA represents a new aspect.

Concerning the prognostic value of initial routine laboratory values, the prognostic value of D-dimers has been described extensively [23,24]. Initial hypercoagulability with the transition to the consumptive stage of disseminated intravascular coagulopathy (DIC) has been reported [25,26,27]. Moreover, Gatto et al. showed a frequent occurrence of pulmonary embolism between days 1 and 47 of hospitalization, occurring in the majority on day 10 [27]. We evaluated the CT images in temporal proximity to the first available blood sampling for the presence of central or distal pulmonal embolism. In those that could be assessed for pulmonary embolism, none were demonstrated. As we used the closest available initial laboratory to identify appropriate treatment predictors, this could explain why pulmonary embolism was not present then. Even Gatto et al. described a high variability of the incidence of pulmonal embolism in COVID-19, thus supporting our result [27]. This may explain why D-dimers were not identified as a marker for predicting the need for intensive care treatment in our study. Still, platelets and PTT were included in the final integrative model emphasizing the importance of hemostaseological diagnostic findings in COVID-19. In addition, models for predicting mortality that combines laboratory or radiological parameters with clinical aspects have already been established via comparable machine-learning approaches [28,29]. Thus, the potential of AI-based algorithms has been demonstrated and can be expanded for interdisciplinary approaches combining laboratory and radiomics values [28]. Predictive endpoints in the previous study by Yu et al. were the need for ventilation and, ultimately, patient mortality. However, our study presents a tool that might help clinicians triage COVID-19 patients upon initial presentation.

For this reason, we have adapted the initial target to predict ICU duration into a categorizing approach and propose a model that might help physicians in emergency departments to distinguish between ICU and normal care demand. Chieregato et al. emphasize that classification into ICU/non-ICU depicts an endpoint representing clinical decision support, a conclusion we would like to emphasize with our results. In addition, the high variation of clinical symptoms was addressed by Chieregato et al. Thus we adopt a purely apparative diagnostic approach [30]. Moreover, we can support this with our results, as there was no significant difference in initial symptom-based severity score between the ICU- and non-ICU cohorts. 

Furthermore, in this study, we augmented routine laboratory parameters used in the mentioned previous studies by cfDNA, a dynamic marker of cell decay. Regarding cfDNA, Cavalier et al. have already described nucleosomal cfDNA to predict requirements for ICU care, underlining the relevance of cfDNA for ICU prediction [5]. We demonstrated significant differences between the cfDNA concentrations in normal and intensive care units (*p* < 0.001), and the importance of predicting ICU demand was verified via the random forest approach.

Moreover, a study by Giraudo et al. presents a radiomics model for predicting ICU transfer [30]. Our results may indicate the elevated diagnostic accuracy of an integrated, multimodal approach for COVID-19 diagnostic evaluation. This can be explained by the additional information offered by routine labs on extrapulmonary organ damage as a compound increased risk of ICU treatment. The results highlight the need for an integrated assessment of interdisciplinary diagnostic data to stratify the planning of treatment capacities better and potentially achieve better clinical outcomes. 

Yet, this study must be interpreted with some limitations. First, the results presented are from a small collective due to many deceased patients during the first global spread of COVID-19, and explicit patient consent was required for cfDNA testing from residual routine care material. Due to the prospective part of the laboratory analysis, not all in-house data could be used for our model, which would have increased the generalizability of our results. In particular, the assessment in a naïve cohort has to be expanded in follow-up studies, as we had to minimize this in favor of the training and validation cohort. Still, we extracted important material and data from the first pandemic waves. We were able to present the applicability of cfDNA and machine-learning algorithms for the stratification of ICU capacities. Thus, this can now be used to extrapolate information from past scenarios that may apply to future variants potentially associated with elevated hospitalization rates again. In a subsequent approach with significantly larger datasets, we aim to evaluate the model’s potential for other SARS-CoV-2 variants. To ensure a higher number of participants, we will assess the necessity of cfDNA in this model. A potential focus on radiomics and routine laboratory parameters could enable its applicability in smaller centers that may not practice these isolation methods and quantification. A purely retrospective analysis would enable the utilization of a larger dataset for testing the model, enhancing the significance, robustness, and generalizability of the potential predictors presented in this study. Additionally, we are considering testing the model on other respiratory diseases to determine whether it is a general model for infectious respiratory diseases or specific to COVID-19.

Considering the selected parameters for the model, further limitations can be discussed. One aspect is the influence of anticoagulatory medication, such as heparin, used in intensive care cohorts. However, when considering the aPTT of both cohorts, no significant difference could be observed, suggesting no influence of heparin on the variable selection. In addition to indicating organ damage, a simultaneous elevation of AST, LDH, and cfDNA may also point to in-vitro hemolysis as a potential confounding factor. To minimize this pre-analytical factor, visual control and photometric evaluation of the sample quality were performed before the analyses ensuring the validity of the results.

Furthermore, there was a certain degree of heterogeneity with regard to the imaging data. Yet, most cases were scanned at one scanner with one standardized protocol. However, a remaining bias cannot be fully ruled out.

With regard to the cfDNA isolation method, it has been shown that the final elution volume and elution steps can be adjusted when the initial plasma volume is low [31]. Since we did not apply these adjustments, this influence on concentration values should be considered when comparing the results with other studies. In addition, a fluorometric approach was used for the concentration measurements, as this is a more cost-effective method and easier to implement in routine diagnostic procedures. In this context, the influence of the carrier RNA contained in the isolation kit may be considered. However, as all samples were treated identically, this does not affect the comparison of our ICU and non-ICU cohorts, ensuring significant differences remain valid. In addition, it is well known that patients with significantly elevated BMI suffer from more severe diseases requiring intensive care treatment [32]. As cfDNA levels are known to correlate with body weight, this might also elevate cfDNA levels in addition to infection-associated cell damage [33]. 

Moreover, pathognomonic symptoms have been included in various other scoring systems [34,35]. Our cohort’s clinical data points of initial symptoms revealed no significant difference among the sub-cohorts. Due to this low discrimination potential, the clinical variables were not included. Furthermore, it could be argued that the pulmonary oxygenation capacity influences clinical disease progression. However, reliable BGA requires arterial blood sampling, which is often difficult to achieve outside intensive care environments, suggesting limited applicability. Equally, including BGA data from non-arterial samples, regularly done during hospital admission, did not provide relevant predictive information for predicting ICU stay. For these reasons, we did not include BGA while still focusing on our model’s radiomics evaluation of lung imaging and the laboratory parameters.

Finally, we established an integrative linear prediction model for the requirement of ICU admission. Based on our cross-validation set, the model was not capable of correctly predicting impending shorter ICU stays of up to 6 days. For this reason, we have resorted to a categorization model of “ICU stay likely/unlikely” with a cut-off at six days of predicted ICU stay. The cut-off is based on the mean deviations of predicted and actual days in ICU in our cross-validation set and was further applied to a validation-naive set with high accuracy. This approach is intended to assist medical staff in assessing the probable demand for intensive treatment during their patients’ initial presentation. The ROC analysis shows a clear superiority of the integrated model compared to the isolated assessment of the biomarkers. We also conclude that cfDNA is a complementary but not essential parameter in this categorized approach. Nevertheless, cfDNA was identified to be significantly elevated in patients requiring intensive care, confirming its potential as a dynamic marker of severe disease.

## 5. Conclusions

In conclusion, we have identified radiomics and laboratory biomarkers associated with a severe COVID-19 course via feature selection algorithms (aim I). The selected parameters have been verified by a random forest approach (aim II), and collinear parameters were reduced via a minimal redundancy algorithm (aim III). Moreover, our results might suggest a solution for a difficult clinical decision-making problem in patients experiencing severe COVID-19, namely, predicting whether a patient on time of admission to the hospital might need ICU treatment shortly. An interdisciplinary approach of integrated diagnostics using laboratory medicine and radiology biomarkers was used to establish this clinical prediction model and was superior to single models (aim IV). Therefore, we propose to study the potentially improved efficiency of ICU capacities using prediction algorithms. Particularly, in scenarios of rapidly rising global infection rates and concomitant hospitalizations, this approach of facilitating triaging vulnerable patients might be relevant.

## Figures and Tables

**Table 1:** Patient collective overview.

	All Patients	Non-ICU Cohort	ICU Cohort	*p* Value
	*n =* 52	*n =* 16	*n =* 36	
Age (mean (SD))	68.46 (13.56)	73.38 (14.94)	66.28 (12.50)	0.081
Gender F/M (%)	22/31 (42.3/57.7)	10/6 (62.5/37.5)	12/25 (33.3/66.7)	0.070
*Symptoms*				
Fever (%)	14 (30.4)	3 (20.0)	11 (35.5)	0.331
Subfebrile (%)	1 (2.2)	1 (6.7)	0 (0.0)	0.326
Night sweat (%)	1 (2.2)	0 (0.0)	1 (3.2)	1.000
Reduced condition (%)	4 (8.7)	2 (13.3)	2 (6.5)	0.587
Diarrhoea (%)	5 (10.9)	2 (13.3)	3 (9.7)	1.000
Cough (%)	16 (34.8)	5 (33.3)	11 (35.5)	1.000
Sore throat (%)	2 (4.3)	0 (0.0)	2 (6.5)	1.000
Dyspnea (%)	16 (34.8)	6 (40.0)	10 (32.3)	0.744
Fatigue (%)	7 (15.2)	2 (13.3)	5 (16.1)	1.000
Nausea (%)	2 (4.3)	0 (0.0)	2 (6.5)	1.000
Anosmia (%)	3 (6.5)	1 (6.7)	2 (6.5)	1.000
Ageusia (%)	4 (8.7)	1 (6.7)	3 (9.7)	1.000
Severity-Score (mean (SD))	1.65 (1.17)	1.60 (1.02)	1.68 (1.26)	0.837
*Treatment*				
ICU days (mean (SD))	9.52 [0.00, 22.66]	0.00 [0.00, 0.00]	16.17 [7.92, 27.06]	**<0.001**
Deceased (%)	3 (5.9)	0 (0.0)	3 (8.6)	0.543
Ventilation (%)	24 (46.2)	0 (0.0)	24 (66.7)	**<0.001**
CVC (%)	28 (53.8)	0 (0.0)	28 (77.8)	**<0.001**
Reanimation (%)	5 (9.6)	0 (0.0)	5 (13.9)	0.308
ICU complex (%)	28 (53.8)	0 (0.0)	28 (77.8)	**<0.001**
*Transfusion*				
erythrocytes/platelets (%)	12 (23.1)	0 (0.0)	12 (33.3)	**0.010**
plasma (%)	1 (1.9)	0 (0.0)	1 (2.8)	1.000
ECMO (%)	6 (11.5)	0 (0.0)	6 (16.7)	0.160
Hemodiafiltration (%)	9 (17.3)	1 (6.2)	8 (22.2)	0.245
Tracheostomy (%)	11 (21.2)	0 (0.0)	11 (30.6)	**0.012**
Operation (%)	9 (17.3)	1 (6.2)	8 (22.2)	0.245
*Laboratory parameters*				
cfDNA (median [IQR]), ng/mL	118.85 [70.58, 292.87]	68.54 [25.73, 93.33]	220.18 [102.19, 25.54]	**<0.001**
Quick (mean (SD)), %	87.75 (17.47)	90.69 (12.32)	86.44 (19.34)	0.424
PTT (median [IQR]), sec.	25.70 [22.03, 34.92]	23.60 [22.17, 26.30]	27.15 [21.65, 38.85]	0.115
D-dimer (median [IQR]), mg/L	1.63 [0.76, 3.90]	1.49 [0.96, 1.73]	1.93 [0.72, 4.06]	0.619
Fibrinogen (mean (SD)), g/L	6.33 (1.91)	5.08 (NA)	6.37 (1.94)	NA
Platelets (mean (SD)), 10^9^/L	270.06 (121.64)	254.25 (130.85)	277.08 (118.57)	0.537
RBC (mean (SD)), 10^12^/L	3.49 (0.71)	3.75 (0.61)	3.38 (0.73)	0.084
Hemoglobin (mean (SD)), g/dL	10.31 (2.18)	10.71 (2.13)	10.14 (2.21)	0.389
MCV (mean (SD)), fl	88.44 (7.36)	84.06 (7.14)	90.38 (6.66)	**0.003**
MCH (median [IQR]), pg	30.10 [28.62, 31.02]	29.05 [27.88, 30.22]	30.65 [29.23, 31.27]	**0.026**
MCHC (mean (SD)), g/dL	33.49 (1.34)	33.92 (1.36)	33.29 (1.31)	0.118
WBC (median [IQR]), 10^9^/L	8.71 [6.26, 11.65]	5.86 [3.92, 8.32]	9.66 [7.94, 15.05]	**<0.001**
CRP (median [IQR]), mg/L	83.50 [41.75, 149.75]	38.50 [27.00, 76.75]	95.55 [64.00, 173.00]	**0.001**
GFR (mean (SD)), mL/min/1.73 m^2^	62.69 (32.35)	62.94 (34.60)	62.58 (31.81)	0.971
Creatinine (median [IQR]), mg/dL	1.03 [0.73, 1.62]	0.92 [0.68, 1.25]	1.03 [0.75, 1.85]	0.258
Urea (median [IQR]), mg/dL	49.10 [37.55, 90.22]	35.60 [30.92, 58.75]	52.45 [41.45, 96.95]	**0.012**
AST (median [IQR]), U/L	38.00 [27.00, 61.00]	29.00 [26.00, 38.00]	48.00 [32.50, 77.75]	**0.016**
ALT (median [IQR]), U/L	32.00 [23.00, 60.00]	26.00 [17.00, 40.00]	39.50 [24.75, 61.50]	0.094
GGT (median [IQR]), U/L	96.00 [37.00, 161.00]	45.00 [28.00, 107.00]	131.00 [38.75, 181.75]	**0.014**
Cholinesterase (mean (SD)), U/L	5722.41 (2140.03)	6459.33 (1974.75)	5588.42 (2169.98)	0.366
Albumin (mean (SD)), g/L	24.13 (5.48)	29.62 (4.02)	21.84 (4.27)	**<0.001**
Bilirubin (median [IQR]), mg/dL	0.41 [0.30, 0.67]	0.44 [0.28, 0.53]	0.40 [0.30, 0.82]	0.464
LDH (median [IQR]), U/L	382.00 [293.50, 447.00]	331.00 [228.00, 389.50]	399.50 [323.50, 469.25]	**0.016**

Presentation of demographic data, initial symptoms, treatment characteristics and laboratory parameters. Non-normally distributed continuous variables were compared by a Kruskal-Wallis rank sum test. For categorical variables, a Fisher exact test was performed. *p*-values < 0.05 were considered significant and are highlighted in bold and underlined.

**Table 2:** Cross-validation of prediction models for routine laboratory parameters and Radiomics.

Internal Cross-Validation I				
*Laboratory Values*	Dataset 1 *Training n = 23 Validation n = 9*	Dataset 2 *Training n = 22 Validation n = 10*	Dataset 3 *Training n = 22 Validation n = 10*	Ranking *frequencies*
partial thromboplastin time	0.364	0.156	0.003	3
Albumin	0.198	0.656	0.679	3
C-reactive protein	0.903	0.712	0.462	3
gamma-glutamyltransferase	0.224	0.517	0.158	3
alanine aminotransferase	0.286		0.163	2
Platelets		0.850	0.806	2
white blood cells	0.375	0.467		2
Urea			0.344	1
glomerular filtration rate			0.065	1
creatinine			0.512	1
red blood cells			0.736	1
mean corpuscular hemoglobin concentration			0.482	1
lactate dehydrogenase	0.495			1
**Internal cross-validation II**				
***Radiomics***	**dataset 1** ***training n = 20 validation n = 10***	**dataset 2** ***training n = 20 validation n = 10***	**dataset 3** ***training n = 20 validation n = 10***	**Ranking *frequencies***
original_firstorder_10Percentile	0.921	0.643	0.508	3
original_gldm_LargeDependenceLowGrayLevelEmphasis	0.236	0.565	0.484	3
original_shape_Maximum2DdiameterSlice	0.355		0.871	2
original_firstorder_Energy	0.707		0.724	2
original_firstorder_TotalEnergy		0.050	0.673	2
original_glcm_ClusterShade	0.299		0.923	2
original_glcm_DifferenceVariance		0.325	0.972	2
original_glrlm_RunEntropy	0.590		0.967	2
original_glrlm_RunLengthNonUniformity	0.891	0.925		2
original_ngtdm_Busyness		0.117	0.493	2
original_ngtdm_Contrast	0.454	0.050		2
original_shape_Elongation		0.277	0.654	2
original_shape_Flatness	0.214	0.387		2
original_shape_LeastAxisLength		0.094	0.462	2
original_shape_MajorAxisLength		0.461	0.961	2
original_shape_Maximum3Ddiameter	0.519		0.823	2
original_firstorder_90Percentile	0.576			1
original_glcm_DifferenceEntropy	0.942			1
original_glcm_MaximumProbability	0.849			1
original_gldm_DependenceNonUniformity	0.905			1
original_gldm_SmallDependenceHighGrayLevelEmphasis	0.235			1
original_glszm_GrayLevelNonUniformity	0.731			1
original_glszm_ZoneEntropy	0.524			1
original_shape_SphericalDisproportion	0.328			1
original_shape_VoxelVolume	0.251			1
original_glcm_ClusterTendency		0.941		1
original_glcm_Imc1		0.254		1
original_glcm_MCC		0.022		1
original_gldm_GrayLevelNonUniformity		0.946		1
original_gldm_SmallDependenceEmphasis		0.577		1
original_glszm_GrayLevelVariance		0.382		1
original_ngtdm_Complexity		0.229		1
original_shape_MinorAxisLength		0.318		1
original_shape_SurfaceVolumeRatio		0.037		1
original_firstorder_Skewness			0.564	1
original_glrlm_GrayLevelNonUniformity			0.726	1
original_glszm_GrayLevelNonUniformityNormalized			0.922	1
original_glszm_LowGrayLevelZoneEmphasis			0.666	1
original_glszm_SizeZoneNonUniformity			0.492	1
original_shape_Compactness1			0.347	1
original_shape_Maximum2DdiameterRow			0.270	1

The difference in the number of subjects in the laboratory (*n* = 32) and radiological (*n* = 30) cross-validation was because two subjects did not receive a chest CT during routine care. *p*-values for the “Laboratory values” and “Radiomics” are presented and variables were excluded from further analysis at a frequency of 1.

**Table 3:** Accuracies of regression models.

Prediction of ICU Demand	
Model	Accuracy
cfDNA	0.47
Radiomics	0.54
Routine lab	0.74
Integrated diagnostics	0.87

## Data Availability

The data presented in this study are available on request from the corresponding author.

## Supplementary Materials

The following supporting information can be downloaded at: https://www.mdpi.com/article/10.3390/microorganisms11071740/s1, Supplemental S1: Blood gas analysis to predict ICU days; Supplemental S2: Verification of reduced features via unsupervised clustering in our validation set.

## Abbreviations

ALAT	Alanine aminotransferase
aPTT	activated partial thromboplastin time
ARDS	Acute respiratory distress syndrome
ASAT	Aspartate aminotransferase
BGA	Blood gas analysis
cfDNA	Circulation free Deoxyribonucleic acid
CAHA	COVID-19-associated hemostatic abnormalities
COVID-19	Coronavirus disease 2019
CRP	C-reactive protein
CT	Computed tomography
DIC	Disseminated intravascular coagulopathy
EDTA	Ethylene diamine tetraacetic acid
GFR	Glomerular filtration rate
GGT	Gamma-glutamyltransferase
ICU	Intensive Care Unit
IL	Interleukin
ISTH	International Society of Thrombosis and Hemostatic
LC	Lymphocyte count
LDH	Lactate dehydrogenase
MCH	Mean corpuscular hemoglobin
MCHC	Mean corpuscular hemoglobin concentration,
MCV	Mean corpuscular volume
MOF	Multiple organ failure
NC	Neutrophil count
NLR	Neutrophil: lymphocyte ratio
PCT	Procalcitonin
PE	Pulmonary embolism
qPCR	quantitative polymerase chain reaction
RSNA	Radiological Society of North America
SARS-CoV-2	Severe acute respiratory syndrome coronavirus type 2
WBC	White blood cells
